# An Integrated Approach to Hygiene, Sanitation, and Storage Practices for Improving Microbial Quality of Drinking Water Treated at Point of Use: A Case Study in Makwane Village, South Africa

**DOI:** 10.3390/ijerph18126313

**Published:** 2021-06-10

**Authors:** Resoketswe Charlotte Moropeng, Phumudzo Budeli, Maggy Ndombo Benteke Momba

**Affiliations:** Department of Environmental, Water and Earth Sciences, Arcadia Campus, Tshwane University of Technology, P/B X 680, Pretoria 0001, South Africa; bphumu@gmail.com (P.B.); mombamnb@tut.ac.za (M.N.B.M.)

**Keywords:** household water treatment systems, sanitation, hygiene

## Abstract

This study assessed the impact of sanitation practices, hygienic and storage conditions on the quality of drinking water treated at point-of-use in Makwane Village. Subsequent to implementation of low-cost Household Water Treatment Devices which are the biosand filter with zeolite-silver (BSZ-SICG) and silver-impregnated porous pot (SIPP) filters in Makwane village, a structured questionnaire was designed to collect the following information: age of caretakers, number of children under the age of five, water storage conditions, sanitation amenities, and hygiene practices. Water quality from the sources to household level was assessed using culture-based and molecular techniques. The results revealed a significant association between the presence of *Escherichia coli* in treated drinking water with the age group of caregivers and the number of children ofless than the age of five [OR (95% CI) = 8.4737 (0.147–3.3497), *p* = 0.0141923 and OR (95% CI) = 9.1667 (0.1848–3.0159); *p* = 0.0165830, respectively]. Moreover, significant association was noted between hygiene practices (washing of hands with/without soap) and water quality in storage containers [OR (95% CI) = 16.000 (0.6763–3.9495), *p* = 0.0000125]. These findings further prove that there is still a dire need for reconsidering hygiene education in rural areas as the health benefits of water treated at point of use (POU) coupled with safe-storage condition interventions might not be guaranteed without proper hygiene. The results further highlighted the importance of washing hands in improving microbial quality of drinking water, which is the key factor for fighting against infectious diseases.

## 1. Introduction

In spite of tremendous progress made worldwide, lack of access to adequate safe drinking water and improved sanitation remains undeniable, particularly in Developing countries. According to the recent reports, over 2.2 billion households lack access to safe drinking water, of which 144 million solemnly depend on surface water [1]. The report further highlight that eight out of ten people without safe drinking water live in rural areas of developing countries. In addition, the report stipulates that 701 million people lack access to improved sanitation with about 673 million people practicing open defecation. 

In addition, some researchers have reported that over 1.3 million death in low- and middle-income areas globally are attributed to unsafe drinking water, inadequate sanitation, and lack of handwashing facilities [2]. The UNICEF 2016 data have revealed that diarrhoea is the second leading killer of children and it accounts for 9% of all deaths among children of less than the age of 5 worldwide [3]. This translates to over 1400 young children dying each day or about 526,000 children a year with 30% of all deaths occurring in Africa [4].

Despite the remarkable achievement made by the democratic governments, access to basic services (safe drinking water and improved sanitation facilities) continue to be one of the most complex challenges facing sub-Saharan African countries, and South Africa is not an exception. The impact of non-standardised or non-existent water treatment and sanitation facilities in public health in under-developed areas of South Africa is enormous. In South Africa, one of the leading causes of morbidity and mortality in children is diarrhoea, accounting for approximately 20% of under-five deaths [5]. Thus, microbial contamination of drinking water poses a health risk to consumers and needs to be controlled effectively. It is a fact that improvements in access to safe Water Sanitation and Hygiene (WaSH), coupled with the promotion of safe storage conditions, can avert childhood diarrhoea [6].

The impact of the scarcity of safe drinking water and sanitation services in Africa falls chiefly on the poor populations, with women and children being the main victims. As a results, women and children are forced to carry water containers over long distances on a daily bases. Moreover, they turned to endure the indignity, shame, and disease that result from a lack of hygiene and sanitation. It has been reported that higher levels of microbial contamination are associated with storage vessels having wide openings (e.g., buckets, plastic drums, and big clay pots), and by dipping drinking vessels (cups/copper or plastic mugs) or unwashed hands into the water storage containers [7]. It is therefore of higher importance to implement effective, affordable, functional, and sustainable intervention strategies for the improvement of the quality of drinking water in such households [8,9]. Moreover, household drinking water storage containers coupled with POU water treatment devices are important in minimizing recontamination of treated stored water in homes [10].

Numerous studies have investigated the behavioral determinants of faecal indicator levels in stored water and on hands being dipped into container-stored water for the identification of the most promising POU interventions for preventing childhood diarrhoea [11,12,13,14]. Consequently, POU water treatment technologies together with safe storage containers have been found to significantly reduce faecal indicator bacteria in stored drinking water at household level [12,13]. In addition, other studies that investigated hand contamination revealed that frequent hand wash with soap also reduces faecal indicator bacteria on hands [14,15]. Furthermore, some studies have shown the associations between faecal indicator bacteria on un-washed hands and in stored drinking water with diarrhoeadiseases in children under 5 years of age [11,16].

Even though washing hand with soap has been shown to reduce the risk of diarrhoea by over 40%, many interventions to improve hand hygiene compliance have been reported to be expensive and can be difficult to scale up and sustain [17,18,19]. Therefore, it is crucial that when interventions of improving the quality of drinking water at POU are made, such circumstances must be taken into consideration. It is also important that households with water treatment devices should be given improved water storage containers which have spigots installed 5–8 cm from the bottom in order to avoid dipping of drinking utensils and hands into the storage containers. This will also reduce the level of faecal indicator bacterial contamination in storage containers. This current study was, therefore, carried out to assess the extent to which sanitation and hygiene practices contribute to the bacterial load recontamination of the drinking water treated at POU and stored in regular and improved containers.

## 2. Materials and Methods

**Figure 1:**
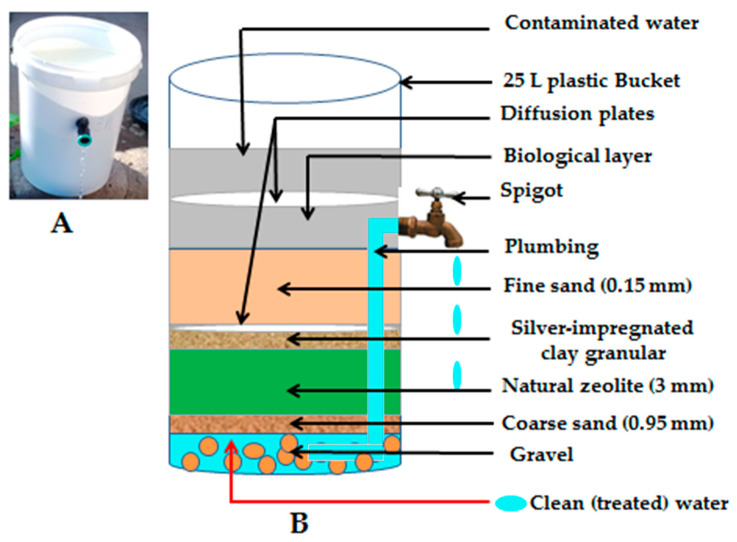
(**A**) Biosand filter with zeolite-silver (BSZ-SICG); (**B**) Schematic representation of the BSZ-SICG. As published by [20].

**Figure 2:**
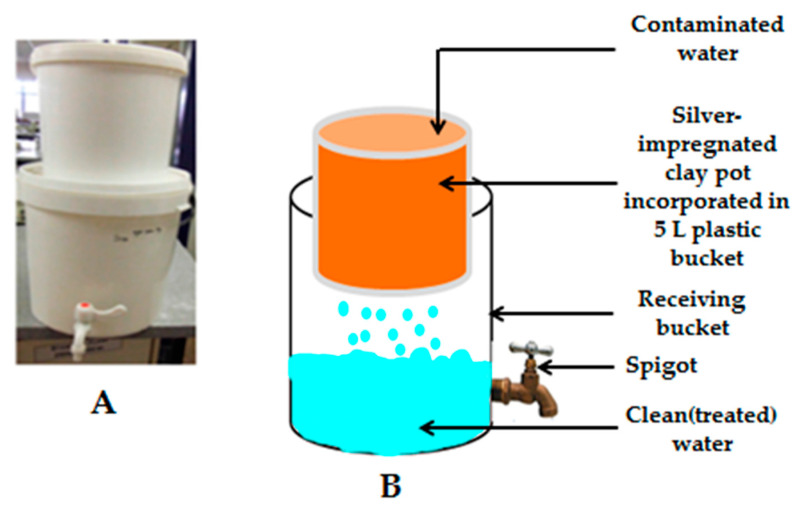
(**A**) Silver-impregnated porous pot (SIPP) filter; (**B**) Schematic representation of the SIPP filter. Adapted from [20].

A cross-sectional study was carried out from May 2015 to November 2015 in Makwane Village households subsequent to implementation of low-cost HWTS interventions, namely biosand filter with zeolite-silver (BSZ-SICG) and silver-impregnated porous pot (SIPP) filters (Figure 1 and Figure 2). A total of 58 households that were utilising the BSZ-SICG and SIPP filters during the study period were randomly selected and followed up for the assessment of the influence of sanitation practices, hygiene, and storage conditions on the quality of drinking water treated at the POU. Households were only qualified for inclusion in this study if the following criteria were met: (1) the primary caregiver was available for interviews; and (2) at least one child under the age of 5 years was living in the household. Confidentiality of all the participants was maintained by assigning a unique code to each of the participants/households.

### 2.1. Description of the Study Area

**Figure 3:**
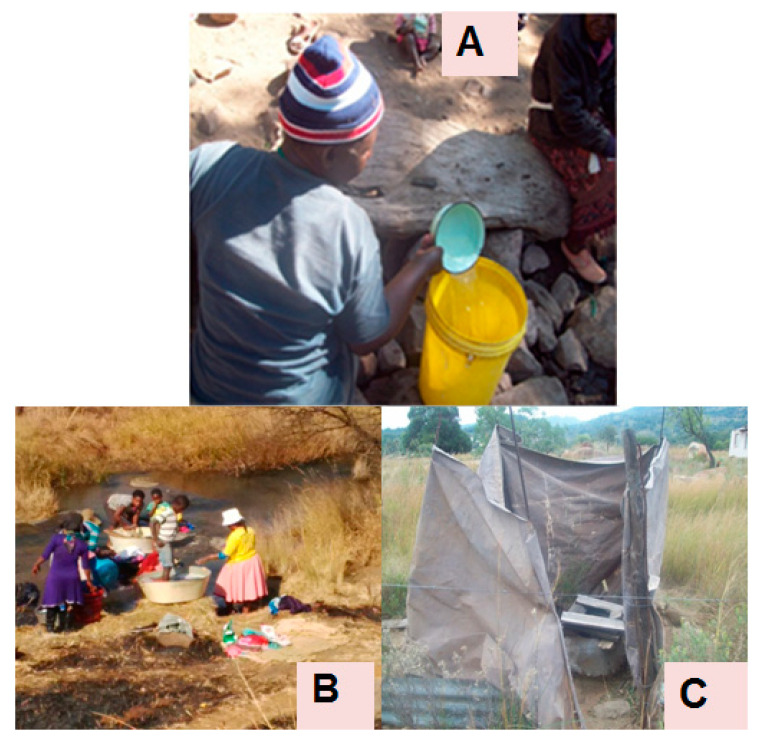
Community member of Makwane collecting water from one of the unprotected sources (**A**); community members doing their laundry in one of the rivers (**B**) where they also collect water for domestic purposes; and (**C**) one of the unsafe pit latrines of Makwane Village.

This current study was conducted in Makwane Village in the Limpopo Province of South Africa. Makwane village is one of the poorest villages in Sekhukhune District, Elias Motsoaledi Municipality. The entire community depends solely on untreated surface water (stream) and springs for domestic purposes and drinking. In addition to lack of safe drinking water, the sanitation facilities are of poor quality and may endanger the health of both elderly and children (Figure 3). Approximately 45.2% of the Makwane community have limited sanitation facilities, of which 13.9% use unimproved facilities. The remaining 40.9% of the community use open defecation.

### 2.2. Ethical Clearance

This study was conducted in accordance with the Declaration of Helsinki, and approved by the Faculty of Science Research Ethics Committee (FCRE) at the Tshwane University of Technology (TUT), where the study was registered (Ref: FCRE 2015/03/040) (2) (SCI). Furthermore, access to the village was obtained through the local pastor and community leaders. All households selected for participation were given informed consent forms at the beginning of the project.

### 2.3. Study Survey

**Figure 4:**
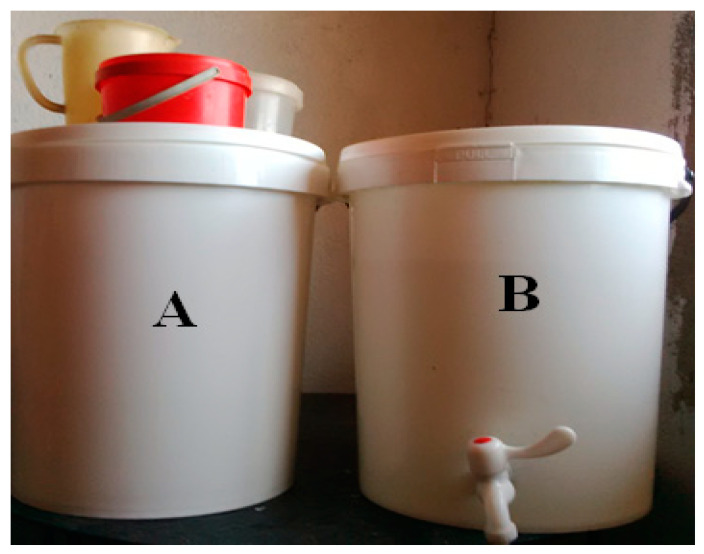
Regular (**A**) and improved (**B**) storage containers supplied to households of Makwane Village.

For the purposes of the study, a structured questionnaire was designed and used to collect information such as number of children under 5 years of age per household, number of individuals living in the household, water sources and storage conditions of the water, sanitation facilities, and hygiene practices. In this study, hygiene practices refer to hand hygiene practices (washing of hands with soap and water after toilet use). Moreover, all selected households were supplied with 2 × 25 L plastic buckets for the storage of treated water. One of the buckets had a spigot installed 5 cm from the bottom (improved storage container) and it was used as intervention of the study, while the other one that did not have a tap installed (regular storage container) was used as the control. Figure 4 shows types of storage containers supplied to the householders.

### 2.4. Water Quality Analysis

A total of 960 water samples from different points (sources, storage/transport containers, hand-rinse samples, drinking vessels, treated water stored in regular containers and treated water in improved containers) were collected between May 2015 and November 2015 and assessed for the presence of pathogenic bacteria using both culture-based and molecular methods. A hand-rinse water sample was taken from children of less than 5 years of age (number of samples depended on the total number of children less than the age of 5) from each participating household. Hand-rinse sampling involved children placing their hands one at a time, into a sterile 1 L sampling plastic bag containing 500 mL of saline water (0.9% *w/v*). For all other samples, a 500 mL polyethylene plastic sterile bottle (RS-Components, Midrend, South Africa) was used. All samples were packed in a cooler box containing ice packs and transported to an in-field laboratory where analyses were performed within 4 h using culture-based techniques. Briefly, spread plate technique was performed for the detection of targeted pathogens. For the isolation of *Vibrio cholerae,* 100 mL of water samples were filtered through 0.22 μm membrane filters. Membranes were subsequently incubated in 100 mL of alkaline peptone water for overnight at 37 °C. Subsequent to incubation, 1 mL of overnight culture broth was streaked on Thiosulfate Citrate Bile agar (TCBS) and incubated for 24 h at 37 °C. For the detection of *E.coli*, *Salmonella* spp. and *Shigella* spp., 3-fold serial dilution was performed after which 1 mL of the samples from the third tube was spread onto MacConkey agar without sorbitol (Merck, Johannesburg, South Africa) for *E. coli* spp., and on Hektoen enteric agar (Merck, SA) for *Salmonella* and *Shigella* species. One loop full of individual presumptive colonies were randomly selected based on their macroscopic characteristics (sizes, shape, and color), and inoculated in 2 mL of brain heart infusion (BHI) broth for overnight at 37 °C. After a period of incubation, the samples were preserved with 20% glycerol. Preserved samples were transported on ice to the Tshwane University of Technology water research group laboratory for further confirmation and analysis.

#### 2.4.1. Genomic DNA Extraction

The DNA from the archived samples was extracted with the ZR Fungal/Bacterial DNA MiniPrep™ test kit (ZYMO Research, Pretoria, South Africa) according to the manufacturer’s procedures. The samples were first purified three times by sub-culturing on nutrient agar followed by incubation at 37 °C overnight.

#### 2.4.2. Molecular Identification of *E. coli*

Amplification of targeted genes was performed in a thermal cycler (MJ Mini^TM^ Personal Thermal Cycler (Bio-RadLaboratories, Johannesburg, South Africa), using published primers listed in Table 1. The polymerase chain reaction (PCR) for identification of the *uidA* gene in *E. coli* that codes for the β-d-glucuronidase enzyme was performed with a 25 µL reaction mixture containing 2.5 µL of template DNA, 12.5 µL of DreamTaq DNA PCR Master Mix (2× DreamTaq Green Buffer, dATP, dCTP, dGTP, and dTTP, 0.4 mM each, and 4 mM MgCl_2_) and 0.18 µL of each primer. Nuclease-free water was added to a final volume of 25 µL. The amplification cycles consisted of an initial DNA denaturation at 95 °C for 4 min, followed by 35 cycles of denaturation at 95 °C for 45 s, primer annealing at 58 °C for 45 s, extension at 72 °C for 1 min, and a final extension at 72 °C for 10 min. Negative controls, substituting the DNA template with nuclease-free water (Inqaba, Pretoria, South Africa) were included in all PCR runs. The DNA extracted from *E. coli* ATCC 25922 (Quantum Biotechnologies, Johannesburg, South Africa) was used as a positive control. The PCR products (8 µL) were visualised with a 1.5% (wt/vol) agarose gel (Life Technologies, Johannesburg, South Africa) at 120 mV for 45 min. A molecular marker (100 bp DNA ladder; Inqaba, Pretoria, South Africa) was run concurrently. All results were captured using a gel documentation system (Syngene, Cambridge, UK).

#### 2.4.3. Molecular Identification of *Salmonella*, *Shigella,* and *V. cholerae*

The DNA templates were subjected to multiplex PCR with specific primers (Table 1) for the detection of the following target genes: invasion plasmid antigen H (*ipaH*) for *Shigella* spp., invasion plasmid antigen B (*ipaB*) for *Salmonella* spp., and superoxide dismutase B (*sodB*) for *V. cholerae*. The amplification cycle conditions consisted of an initial denaturation at 95 °C for 15 min, followed by 35 cycles of denaturation at 94 °C for 45 s, annealing at 57 °C for 45 s, and extension at 72 °C for 1 min. This was followed by a final extension step at 72 °C for 5 min. The DNA extracted from *Salmonella* ATCC 14028, *Shigella* ATCC 11835 and *V. cholerae* ATCC 25920 (Quantum Biotechnologies, Johannesburg, South Africa) was used as a positive control. The PCR products (8 µL) were visualised with a 1.5% (*w*/*v*) agarose gel (Life Technologies, Johannesburg, South Africa) at 120 mV for 60 min. A molecular marker (1 kb DNA ladder; Inqaba, Pretoria, South Africa) was run concurrently. All results were captured using a gel documentation system (Syngene, Cambridge, UK).

### 2.5. Statistical Analysis

*E. coli* was used as an indicator of recent faecal contamination in this study. Conditional logistic regression was used to calculate the matched odds ratio (OR) representing the association between demographic characteristics, sanitation, hygiene, and the presence of *E. coli* in the households’ stored treated drinking water (stored in regular containers). Exact *p*-values and 95% confidence intervals (CI) were calculated.

## 3. Results

### 3.1. Socio-Demographic Information of the Study Area

An overview of socio-demographic information of Makwane Village obtained subsequent to HWTS implementation is presented in Table 2. The survey revealed that the majority of the caregivers were aged between 32 to 36 years (31.0%), followed by age group of 22–26 (27.6%) and 27–31 years (24.1%). Out of 58 households surveyed, 22 (37.9%) were found to have at least three children under 5 years of age, while 4 (6.9%) households had four children in this age group. Most of the households (43.1 %) have 5 to 7 members in the house. The majority of households (51.7%) used open pit latrines as a sanitation facility and 43.1% of the households used the open field as an alternative, while 5.2% relieved themselves in streams/rivers. The findings of the current study also revealed that 72.4% of the households surveyed generally depend on the river/stream as their source of water for domestic use and other purposes. The hygiene practices (washing of hands with/without soap after using toilet) in the households were also assessed and it was revealed that 93.1% of the household members do not wash their hands after relieving themselves. Only 6.9% of the surveyed households reported that they wash their hands without soap after using toilet. All of the surveyed households (100%) were equipped with and used HWTS devices for treating water at POU.

### 3.2. Overall Bacteriological Quality of Makwane Households’ Drinking Water

**Figure 5:**
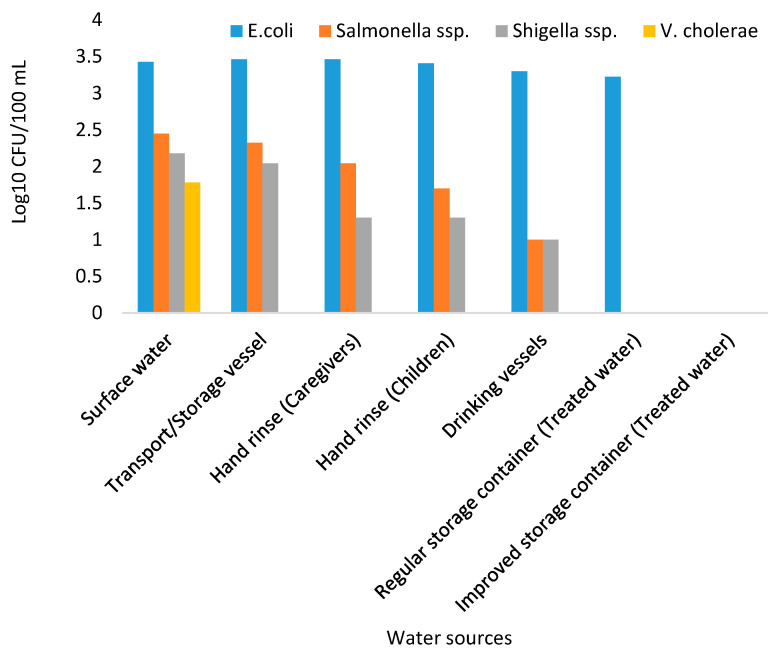
Mean concentration (Log10 CFU/100 mL) of presumptive enteropathogenic bacteria from different water sources in Makwane Village.

During the study period, a total of 960 water samples collected from surface water sources, storage containers, drinking vessels, regular storage containers, improved storage containers, and hand-rinse water of caregivers and children under the age of 5 years were analysed using culture-based methods for the presence of presumptive enteropathogenic bacteria. The mean concentrations of presumptive enteropathogenic bacteria are presented in Figure 5. Of all the enteropathogenic bacteria isolated, *E. coli* was found to have higher counts followed by *Salmonella, Shigella,* and *V. cholerae,* respectively. Moreover, none of the enteropathogenic bacteria were detected in treated water stored in improved containers. Of all the enteropathogenic bacteria enumerated, *E. coli* was found to be more prevalent with the counts ranging from 3.462 Log10 CFU/100 mL to 3.225 Log10 CFU/100 mL, followed by *Salmonella* spp. and *Shigella* spp. with counts ranging from 2.447 Log10 CFU/100 mL to 1 Log10 CFU/100 mL and 2.176 Log10 CFU/100 mL to 1 Log10 CFU/100 mL, respectively. *Vibrio* spp. was the least detected, with their counts ranging from 0 to 1.778 Log10 CFU/100 mL and it was only detected in surface water samples.

### 3.3. Confirmation of Enteropathogenic Bacteria by PCR

**Figure 6:**
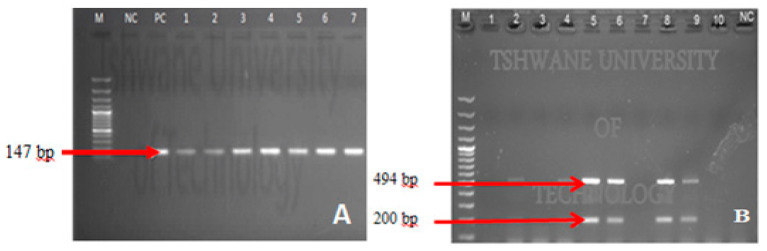
Gel electrophoresis of confirmed enteropathogenic bacteria; Figure 6A shows an agarose gel picture of results obtained from conventional PCR in detection of uidA gene for *E. coli* (147 bp) while Figure 6B shows an agarose gel picture of results obtained from multiplex PCR in detection of *ipaB* (494 bp) and *ipaH* (200 bp) genes for *Salmonella* spp. and *Shigella* spp., respectively. M: Molecular Marker ((**A**) 100 bp and (**B**) 1 kb); Lane 1–10 test organism; NC: Negative Control; PC: Positive Control.

Archived presumptive bacterial isolates were further confirmed by conventional PCR. All target genes were confirmed with the exception of the *sodB* gene for *V. cholerae*. Figure 6 shows some of the PCR products on the agarose gel after gel-electrophoresis.

### 3.4. Geometric Mean Concentration of Presumptive E. coli in Treated Water Stored in Regular Containers Relative to Socio-Demographic Characteristics

**Figure 7:**
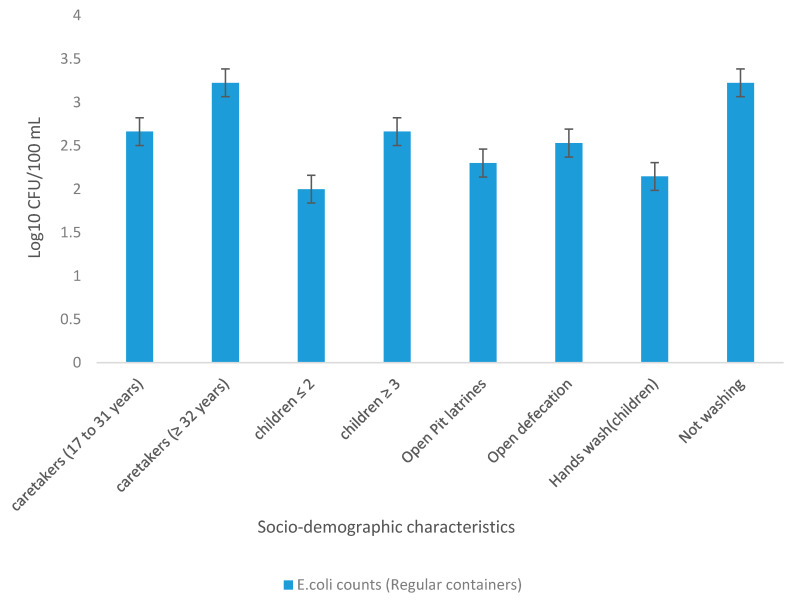
Mean concentration of *E. coli* (Log10 CFU/100 mL) in treated water collected from regular storage container versus socio-demographic characteristics.

Households were grouped according to socio-demographic characteristics to determine the mean concentration of presumptive *E. coli* in treated drinking water stored in regular containers. The results are presented in Figure 7. The results revealed higher counts of *E. coli* in households with caregivers aged 32 years and above (3.23 Log10 CFU/100 mL) and lower counts in households with caregivers aged between 17 and 31 years (2.66 Log10 CFU/100 mL). An association between the number of children less than 5 years of age in the households and the quality of water stored in regular containers was also shown. Water samples of households with two or fewer children under the age of 5 years were shown to have lower *E. coli* counts (2.0 Log10 CFU/100 mL), while water samples in households with 3 and more children were found to have higher *E. coli* counts (2.67 Log10 CFU/100 mL). The *E. coli* counts in water samples of households that used open pit latrines were found to be lower (2.30 Log10 CFU/100 mL) as opposed to those of households that did not have pit latrines (2.531 Log10 CFU/100 mL). Very low *E. coli* counts (2.146 Log10 CFU/100 mL) were observed in water storage containers of households that were practising proper hand hygiene (washing of hands after using the toilet) while higher *E. coli* counts (3.225 Log10 CFU/100 mL) were observed in water storage containers of those that did not practise proper hygiene.

### 3.5. Relationship between Demographic Information and Water Quality in Terms of E. coli Mean Concetration in Treated Water Stored in Regular Containers

The relationship between mean concentration of *E. coli* isolates in stored water samples and age group of caregivers together with the total number of children under the age of 5 years in the households is illustrated in Table 3. The results showed a significant association between the presence of *E. coli* in treated drinking water stored in regular containers and the age group of caregivers [OR (95% CI) = 8.47 (0.15–3.35)]. There was also a significant association between the presence of *E. coli* in treated drinking water stored in regular containers and the total number of children under the age of 5 years in the households (OR (95% CI) = 9.17 (0.18–3.02)).

Further analyses were performed to determine the relationship between sanitation, hygiene practices, and the quality of treated water stored in regular containers. *Escherichia coli* was used as an indicator of recent faecal contamination in this study. The results are presented in Table 3. The results revealed a relationship between the presence and absence of sanitation facilities with water quality in terms of *E. coli* counts in treated drinking water stored in regular containers (OR (95% CI) = 0.91 (−0.97–0.88), but the relationship was not significant. On the other hand, results showed a significant relationship between treated water stored in regular containers and hygiene practice (OR (95% CI) = 16.00 (0.68–3.95)).

## 4. Discussion

Scarcity of water coupled with poor sanitation, substandard water quality, and inappropriate hygiene behaviour are disastrous for infants and young children. As a result, children under the age of 5 years die each year due to diarrhoea diseases caused by ingestion of contaminated water [25,26]. This study investigated the microbiological quality of water used by the rural population of Makwane Village in relation to storage conditions, sanitation, and hygiene practices. The results of this study revealed that both water source (surface water) and household water samples (treated water in regular containers) had higher bacterial counts, which exceeded the recommended limits as set out in the WHO guidelines for water intended for household purposes [27]. The presence of *E. coli, Salmonella,* and *Shigella* in the water samples implies that there is a high risk of afaecalfaecal contamination of water and that consequently users are at risk of becoming ill from microbial pathogens of faecal origin [27,28]. A study by previous investigators [29] stated that if source water is of poor microbial quality prior to collection, transportation, and storage, then the quality of this water is likely to deteriorate even further due to external contaminant exposure. This was the case in this study as it was found that the bacterial counts at source and in storage containers (before treatment) were extremely high and the water quality deteriorated in regular containers after treatment (Figure 5). The presumptive bacteria were further confirmed by conventional PCR (Figure 6). The presence of enteropathogenic bacteria in treated water stored in regular containers indicated faecalfaecal contamination, which could be attributed to poor personal hygiene as it was indicated in the survey that 93.1% of the community never washed their hands after using the toilet (Table 2).

Previous studies have reported the relationship between the microbial deterioration of drinking water quality at the POU and the presence of children of less than 5 years of age in the households [30,31]. Nonetheless, few studies have investigated the link between POU water quality and the number of children under the age of 5 years in households. Moreover, children have been shown to contribute significantly to faecalfaecal contamination of drinking water through insertion of hands into storage containers when drawing water [28,31,32]. Playing near storage vessels can also contribute to the deterioration of treated drinking water quality through dust and sand particles that can enter the uncovered storage vessels [32,33]. In this study, the link between water quality and the number of children under the age of 5 was investigated and it was found that households with 1 to 2 children of less than 5 years old had lower *E. coli* counts as opposed to households with 3 and more children under the age of 5 years (Figure 7). These findings indicate that the number of children less than 5 years old in the household significantly contribute to faecalfaecal contamination of water in storage containers at homes. Therefore, it is very crucial for the caregivers to make sure that children do not play next to water storage containers as this results in the deterioration of drinking water and could pose a very serious health problem to the end users.

Not only does the age and number of children contribute to microbial deterioration of drinking water, but also the age of elders/caregivers contributes to deterioration of water quality at POU [32]. In this study, the link between age of caregivers and water quality was investigated. It was observed that the age of household members was associated with the quality of stored drinking water in regular containers. Households with caregivers aged 32 years and above were found to have higher *E. coli* counts (3.23 Log10 CFU/100 mL) as opposed to households with caregivers aged between 17 and 31 years (2.66 Log10 CFU/100 mL). Sanitation and hygiene practices are likely to have made contributions to microbial drinking water quality at POU in this study (Figure 7). It was demonstrated that households which practised open defecation had the highest levels of *E. coli* (2.531 Log10 CFU/100 mL) in drinking water at the point-of-use, while households with pit latrines had lower *E. coli* counts (2.301 Log10 CFU/100 mL), but the difference was not significant. Lack of proper hygiene practices, such as drawing water by dipping utensils into storage vessels and not washing hands after using the toilet, has been shown to contribute to the deterioration of microbial quality of drinking water [34,35].

In addition, good hygiene practices, especially hand washing, were previously shown to be an effective intervention in the reduction of waterborne diseases in developing countries [36]. Another study by previous investigators [15] showed that faecalfaecal contamination from children and adults who do not wash their hands after using the toilet can contribute to secondary contamination of household stored drinking water. The same authors have also indicated that *E. coli* can survive for 10 min and *Shigella sonnei* for up to 3 h on unwashed hands, which can contaminate water and food in the household [15]. The results of this current study support the findings of a previous study [15] that also highlighted lower *E. coli* counts in treated stored drinking water of members who washed their hands after using the toilet. In contrary, the study revealed that treated stored drinking water in households with members not washing their hands exhibited higher *E. coli* counts (3.225 Log10 CFU/100 mL) in water at POU (Figure 7). These findings further prove that hands serve as a pathway for the transmission of pathogenic bacteria in treated drinking water. Therefore, washing of hands after using toilet is essential as it has been shown to reduce bacterial contaminants in water at POU.

This current study also investigated the association between microbial quality of drinking water at POU with demographic information (age of caretakers and number of children less than the age of 5 years) per households. A significant association (OR (95% CI) = 8.47 (0.15–3.35)) between age of the caregivers and water quality was noted in this study (Table 3). This finding implies that households with caregivers aged ≥ 32 years are at risk of being exposed to *E. coli* in their treated water stored in regular containers as opposed to households with caregivers aged between 17 and 31 years. This could be due to the fact that most of the caregivers aged ≥32 years were found not to wash their hands after changing nappies or after using the toilet. Number of children in the households also showed a significant association with water quality in terms of *E. coli* in water stored in regular containers at POU (OR (95% CI) = 9.17 (0.18–3.02). These findings indicate that households with 3 or more children under the age of 5 years are at higher risk of being exposed to pathogenic bacteria if treated water is stored in regular containers. Moreover, the findings of this current study also showed that children under the age of 5 years in Makwane Village are at risk of waterborne diseases. Therefore, the use of improved storage containers should be regarded as an important intervention and should be mandatory in this village in order to reduce the deterioration of water quality at POU.

The quality of water at POU in association with sanitation and hygiene practices was also investigated in this study. Previous studies have shown that the type of sanitation used, i.e., urine diversion toilets, ventilated improved pit toilets, unimproved open pit toilets, or open defecation also contributes to poor microbial quality of drinking water at POU and increases the health outcomes such as diarrhoea [28,31,36]. In this study, a significant association [OR (95% CI) = 16.00 (0.68–3.95)] between hygiene and water quality in terms of *E. coli* counts in regular storage containers was revealed (Table 3). These observations imply that household members who never wash their hands after using the toilet are at higher risk of being exposed to waterborne pathogens in the storage containers as opposed to household members who wash their hands after using the toilet. Furthermore, this study has shown the importance of washing hands after using the toilet. In addition, the results of this study showed a relationship between sanitation and water quality [OR (95% CI) = 0.91 (−0.97–0.88)] although the relationship was not significant. The relationship between sanitation and the quality of drinking water might have been attributed to lack of improved sanitation facilities in Makwane Village as the entire village depends on open pit latrines (Figure 3C). Although the use of the BSF-ZS and SIPP filters (treated water stored in improved containers) was shown to improve the quality of the drinking water (Figure 7), recontamination at the point of consumption significantly reduced the quality of this water. Some of the factors that contributed to the recontamination at POU could be a lack of proper sanitation and immersion of contaminated hands in storage containers. In addition, the formation of biofilm on the sidewalls of storage containers is another contributing factor to recontamination of treated water at POU as it causes regrowth of microorganisms [37]. Furthermore, previous investigators [38] have also shown that organisms can prosper in biofilms in storage containers. Therefore, it is also crucial to wash the storage containers regularly to avoid the formation of biofilms.

### The Relevance of the Obtained Findings to the Current Situation

In a study conducted by Moropeng et al., 2018, it was noted that the life span of the water treatment systems implemented in Makwane village was 6 months for Biosand filters with zeolite and silver impregnated granular clay and 12 month for the silver-impregnated porous pot filters. Since this was a pilot study, lack of funding to sustain the water treatment devices has led the community reverting to old ways of relying on untreated water sources as they would prior to implementation. The main objective of the study was to assess the impact of sanitation practices and hygienic and storage conditions on the quality of drinking water treated at point-of-use in Makwane Village. Thus, after the systems have exhausted their lifespan, the investigation was halted due to unavailability of treated water samples. Nonetheless, the findings obtained from this study suggest that although the implemented systems were efficacious in removing waterborne pathogens resulting from poor hygiene practice, its implementation as a stand-alone intervention may not be efficacious in combating WaSH associated disease. Therefore, this study submits that the implementation of such systems should always be conducted in conjunction with knowledge of good hygiene and sanitation practice to avoid recontamination of treated water, which inevitably reverses their health benefits. The importance of access to clean water and good hygiene practices under normal circumstance and particularly during the COVID-19 pandemic cannot be over-emphasized (Budeli et al., 2021). Furthermore, the implementation of these water treatment systems will not only aid in producing clean water but also mitigate the economic burden which is worsened by the current lockdown. Since these systems can be manufactured at a low cost (below $20), they present a viable option for drinking water treatment during the current pandemic and beyond, especially in secluded areas that are less likely to receive centralized water channels in the near future.

## 5. Conclusions and Recommendations

The increase in contamination of treated drinking water stored in regular containers may be as a result of poor water handling within households due to such things as dipping of hands inside the storage containers during water collection, especially when hands are not washed after using the toilet. The findings of the current study prove that there is still a need to reconsider hygiene education in rural areas of South Africa as the health benefits of HWTS interventions might not be guaranteed without proper hygiene and sanitation practices. Consequently, this study recommends the use of improved storage containers and training of caregivers on strategies for safe storage, handling, and hygiene conditions within households in order to improve drinking water quality in dwellings.

## Figures and Tables

**Table 1:** Oligonucleotides used in this study for amplification of housekeeping genes for *E. coli, Shigella, Salmonella,* and *Vibrio* spp.

Pathogen	Gene Targeted/Oligonucleotide Name	Sequence (5′-3′)	Gene Size (bp)	Ref.
*E. coli*	*uidA*-F	-AAAACGGCAAGAAAAAGCAG-	147	[21]
	*uidA*-R	-ACGCGTGGTTAACAGTCTTGCG-		
*Shigella*	*ipaH*-F	-GTTACCTGTACTCCCTGCTT-R CTAGCCTTCCTTGTGCAA	200	[22]
	*ipaH*-R	-CTAGCCTTCCTTGTGCAA-		
*Salmonella*	*ipaB*-F	-GGACTTTTTAAAAGCGGCGG-	494	[23]
	*ipaB*-R	-GCCTCTCCCAGAGCCGTCTGG-		
*Vibrio*	*sodB*-F	-AAGACCTCAACTGGCGGTA-	248	[24]
	*sodB*-R	-GAAGTGTTAGTGATCGCCAGAGT-		

**Table 2:** Socio-demographic data of Makwane households surveyed subsequent to implementation of HWTS technology.

Characteristics	Frequency N = 58	Percentage %
Caregivers per age group		
17–21	3	5.2
22–26	16	27.6
27–31	14	24.1
32–36	18	31
≥37	7	12.1
Number of children < 5 years		
1	15	25.9
2	17	29.3
3	22	37.9
4	4	6.9
Number of household members		
2–4	19	32.8
5–7	25	43.1
8–9	12	20.7
≥10	2	3.4
Sanitation facilities		
Open pit latrines	30	51.7
Open field defecation	25	43.1
Defecate in streams	3	5.2
Water sources		
Surface water	42	72.4
Spring water	4	6.9
Borehole water	12	20.7
Hygiene		
Wash hands after using toilet		
With soap	0	0
Without soap	14	6.9
Never wash hands after using toilet	44	93.1

**Table 3:** Relationship between demographic characteristics, socio-demographic characteristics, and mean concentration of *E. coli* isolated from treated water stored in regular containers. *N* = 58.

Demographic Characteristics		*E. coli* Counts (<1 Log10 CFU/100 mL)	*E. coli* Counts (≥1 Log10 CFU/100 mL)	OR (95% CI)	*p*-Value
Age groups of caregivers	17–31	14 (42.4%)	19 (57.6%)	8.47 (0.15–3.35)	<0.05
	≥32	2 (8%)	23 (92%)		
Number of children < 5 years per household	≤2	20 (62.5)	12 (37.5)	9.17 (0.18–3.02)	<0.05
	≥3	4 (11.5)	22 (88.5)		
Sanitation facility	Open pit latrines	8 (26.7)	22 (73.3)	0.91 (−0.97–0.88)	>0.5
	Open defecation	8 (28.6)	20 (71.4)		
Hygiene practice	Wash hands	12 (85.7)	2 (14.3)	16.00 (0.68–3.95)	<0.05
	Do not wash hands (with/without soap)	12 (27.3)	32 (72.7)		

N: Sample size; OR: Odds ratio; 95% CI: confidence interval. *N* = 58.
